# The human body at cellular resolution: the NIH Human Biomolecular Atlas Program

**DOI:** 10.1038/s41586-019-1629-x

**Published:** 2019-10-09

**Authors:** HuBMAP Consortium, Michael P. Snyder, Shin Lin, Amanda Posgai, Mark Atkinson, Aviv Regev, Jennifer Rood, Orit Rozenblatt-Rosen, Leslie Gaffney, Anna Hupalowska, Rahul Satija, Nils Gehlenborg, Jay Shendure, Julia Laskin, Pehr Harbury, Nicholas A. Nystrom, Jonathan C. Silverstein, Ziv Bar-Joseph, Kun Zhang, Katy Börner, Yiing Lin, Richard Conroy, Dena Procaccini, Ananda L. Roy, Ajay Pillai, Marishka Brown, Zorina S. Galis, Long Cai, Jay Shendure, Cole Trapnell, Shin Lin, Dana Jackson, Michael P. Snyder, Garry Nolan, William James Greenleaf, Yiing Lin, Sylvia Plevritis, Sara Ahadi, Stephanie A. Nevins, Hayan Lee, Christian Martijn Schuerch, Sarah Black, Vishal Gautham Venkataraaman, Ed Esplin, Aaron Horning, Amir Bahmani, Kun Zhang, Xin Sun, Sanjay Jain, James Hagood, Gloria Pryhuber, Peter Kharchenko, Mark Atkinson, Bernd Bodenmiller, Todd Brusko, Michael Clare-Salzler, Harry Nick, Kevin Otto, Amanda Posgai, Clive Wasserfall, Marda Jorgensen, Maigan Brusko, Sergio Maffioletti, Richard M. Caprioli, Jeffrey M. Spraggins, Danielle Gutierrez, Nathan Heath Patterson, Elizabeth K. Neumann, Raymond Harris, Mark deCaestecker, Agnes B. Fogo, Raf van de Plas, Ken Lau, Long Cai, Guo-Cheng Yuan, Qian Zhu, Ruben Dries, Peng Yin, Sinem K. Saka, Jocelyn Y. Kishi, Yu Wang, Isabel Goldaracena, Julia Laskin, DongHye Ye, Kristin E. Burnum-Johnson, Paul D. Piehowski, Charles Ansong, Ying Zhu, Pehr Harbury, Tushar Desai, Jay Mulye, Peter Chou, Monica Nagendran, Ziv Bar-Joseph, Sarah A. Teichmann, Benedict Paten, Robert F. Murphy, Jian Ma, Vladimir Yu. Kiselev, Carl Kingsford, Allyson Ricarte, Maria Keays, Sushma A. Akoju, Matthew Ruffalo, Nils Gehlenborg, Peter Kharchenko, Margaret Vella, Chuck McCallum, Katy Börner, Leonard E. Cross, Samuel H. Friedman, Randy Heiland, Bruce Herr, Paul Macklin, Ellen M. Quardokus, Lisel Record, James P. Sluka, Griffin M. Weber, Nicholas A. Nystrom, Jonathan C. Silverstein, Philip D. Blood, Alexander J. Ropelewski, William E. Shirey, Robin M. Scibek, Paula Mabee, W. Christopher Lenhardt, Kimberly Robasky, Stavros Michailidis, Rahul Satija, John Marioni, Aviv Regev, Andrew Butler, Tim Stuart, Eyal Fisher, Shila Ghazanfar, Jennifer Rood, Leslie Gaffney, Gokcen Eraslan, Tommaso Biancalani, Eeshit D. Vaishnav, Richard Conroy, Dena Procaccini, Ananda Roy, Ajay Pillai, Marishka Brown, Zorina Galis, Pothur Srinivas, Aaron Pawlyk, Salvatore Sechi, Elizabeth Wilder, James Anderson

**Affiliations:** 1 Department of Genetics, Stanford School of Medicine, Stanford, CA USA; 2 Department of Medicine, University of Washington, Seattle, WA USA; 3 Department of Pathology, University of Florida Diabetes Institute, Gainesville, FL USA; 4 Klarman Cell Observatory Broad Institute of MIT and Harvard, Cambridge, MA USA; 5 Howard Hughes Medical Institute, Koch Institute of Integrative Cancer Research, Department of Biology, Massachusetts Institute of Technology, Cambridge, MA USA; 6 New York Genome Center, New York, NY USA; 7 New York University, New York, NY USA; 8 Department of Biomedical Informatics, Harvard Medical School, Boston, MA 02115 USA; 9 Brotman Baty Institute for Precision Medicine, Allen Discovery Center for Cell Lineage Tracing, Howard Hughes Medical Institute, Department of Genome Sciences, University of Washington, Seattle, WA USA; 10 Department of Chemistry, Purdue University, West Lafayette, IN USA; 11 Department of Biochemistry, Stanford University School of Medicine, Stanford, CA USA; 12 Pittsburgh Supercomputing Center, Carnegie Mellon University, Pittsburgh, PA USA; 13 Department of Biomedical Informatics, University of Pittsburgh, Pittsburgh, PA USA; 14 Computational Biology Department, School of Computer Science, Carnegie Mellon University, Pittsburgh, PA USA; 15 Department of Bioengineering, University of California San Diego, La Jolla, CA USA; 16 Department of Intelligent Systems Engineering, School of Informatics, Computing, and Engineering, Indiana University, Bloomington, IN USA; 17 Department of Surgery, Washington University School of Medicine, St Louis, MO USA; 18 Office of Strategic Coordination, Division of Program Coordination, Planning, and Strategic Initiatives, National Institutes of Health, Bethesda, MD USA; 19 National Human Genome Research Institute, National Institutes of Health, Bethesda, MD USA; 20 National Heart, Lung, and Blood Institute, National Institutes of Health, Bethesda, MD USA; 21 Department of Biology and Biological Engineering, California Institute of Technology, Pasadena, CA USA; 22 Department of Microbiology, Stanford School of Medicine, Stanford, CA USA; 23 Department of Radiology, Stanford School of Medicine, Stanford, CA USA; 24 Department of Medicine, Washington University in St Louis, St Louis, MO USA; 25 Department of Pediatrics, University of North Carolina School of Medicine, Chapel Hill, NC USA; 26 Department of Pediatrics, University of Rochester, Rochester, NY USA; 27 Institute of Molecular Life Sciences, University of Zurich, Zurich, Switzerland; 28 Department of Neuroscience, University of Florida, Gainesville, FL USA; 29 Department of Biomedical Engineering, University of Florida, Gainesville, FL USA; 30 Mass Spectrometry Research Center, Department of Biochemistry, Vanderbilt University, Nashville, TN USA; 31 Department of Medicine, Vanderbilt University Medical Center, Nashville, TN USA; 32 Department of Pathology, Microbiology and Immunology, Vanderbilt University Medical Center, Nashville, TN USA; 33 Delft Center for Systems and Control, Delft University of Technology, Delft, The Netherlands; 34 Department of Cell and Developmental Biology, Vanderbilt University, Nashville, TN USA; 35 Department of Biostatistics and Computational Biology, Dana-Farber Cancer Institute, Boston, MA USA; 36 Wyss Institute for Biologically Inspired Engineering, Harvard University, Boston, MA USA; 37 Department of Systems Biology, Harvard Medical School, Boston, MA USA; 38 Department of Electrical and Computer Engineering, Opus College of Engineering, Marquette University, Milwaukee, WI USA; 39 Biological Sciences Division, Pacific Northwest National Laboratory, Richland, WA USA; 40 Department of Internal Medicine, Division of Pulmonary & Critical Care, Stanford University School of Medicine, Stanford, CA USA; 41 Cellular Genetics Programme, Wellcome Sanger Institute, Hinxton, UK; 42 Department of Biomolecular Engineering, Jack Baskin School of Engineering, University of California Santa Cruz, Santa Cruz, CA USA; 43 Opto-Knowledge Systems, Torrance, CA USA; 44 Department of Biology, University of South Dakota, Vermillion, SD USA; 45 Renaissance Computing Institute, University of North Carolina, Chapel Hill, NC USA; 46 Department of Genetics, University of North Carolina, Chapel Hill, NC USA; 47 School of Information and Library Science, University of North Carolina, Chapel Hill, NC USA; 48 Knowinnovation, Buffalo, NY USA; 49 European Molecular Biology Laboratory, European Bioinformatics Institute (EMBL-EBI), Wellcome Genome Campus, Hinxton, UK; 50 Cancer Research UK Cambridge Institute, University of Cambridge, Cambridge, UK; 51 National Institute of Diabetes and Digestive and Kidney Diseases, National Institutes of Health, Bethesda, MD USA

**Keywords:** Computational platforms and environments, Genomics

## Abstract

Transformative technologies are enabling the construction of three-dimensional maps of tissues with unprecedented spatial and molecular resolution. Over the next seven years, the NIH Common Fund Human Biomolecular Atlas Program (HuBMAP) intends to develop a widely accessible framework for comprehensively mapping the human body at single-cell resolution by supporting technology development, data acquisition, and detailed spatial mapping. HuBMAP will integrate its efforts with other funding agencies, programs, consortia, and the biomedical research community at large towards the shared vision of a comprehensive, accessible three-dimensional molecular and cellular atlas of the human body, in health and under various disease conditions.

## Abstract (web summary)

HuBMAP supports technology development, data acquisition, and spatial analyses to generate comprehensive molecular and cellular three-dimensional tissue maps.

## Main

The human body is an incredible machine. Trillions of cells, organized across an array of spatial scales and a multitude of functional states, contribute to a symphony of physiology. While we broadly know how cells are organized in most tissues, a comprehensive understanding of the cellular and molecular states and interactive networks resident in the tissues and organs, from organizational and functional perspectives, is lacking. The specific three-dimensional organization of different cell types, together with the effects of cell–cell and cell–matrix interactions in their natural milieu, have a profound impact on normal function, natural ageing, tissue remodelling, and disease progression in different tissues and organs. Recently, new technologies have enabled the molecular characterization of a multitude of cell types^1–4^ and mapping of their spatial relationships in complex tissues at unprecedented scale and single-cell resolution. These advances create the opportunity to build a high-resolution atlas of three-dimensional maps of human tissues and organs.
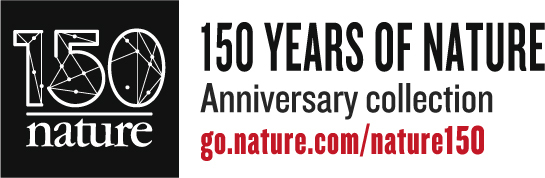


HuBMAP (https://commonfund.nih.gov/hubmap) is an NIH-sponsored program with the goals of developing an open framework and technologies for mapping the human body at cellular resolution as well as generating foundational maps for several tissues obtained from normal individuals across a wide range of ages. A previous NIH-sponsored project, GTEx^5^, examined DNA variants and bulk tissue expression patterns across approximately a thousand individuals, but HuBMAP is a distinct project focused on generating molecular maps that are spatially resolved at the single-cell level but using samples from a more limited number of people. To achieve these goals, HuBMAP has been designed as a cohesive and collaborative organization, with a culture of openness and sharing using team science-based approaches^6^. The HuBMAP Consortium (https://hubmapconsortium.org/) will actively work with other ongoing initiatives including the Human Cell Atlas^7^, Human Protein Atlas^8^, LIfeTime (https://lifetime-fetflagship.eu/), and related NIH-funded consortia that are mapping specific organs (including the brain^9^, lungs (https://www.lungmap.net/), kidney (https://kpmp.org/about-kpmp/), and genitourinary (https://www.gudmap.org/) regions) and tissues (especially pre-cancer and tumours^10^; https://humantumoratlas.org), as well as other emerging programs.

## HuBMAP organization and approaches

**Fig. 1:**
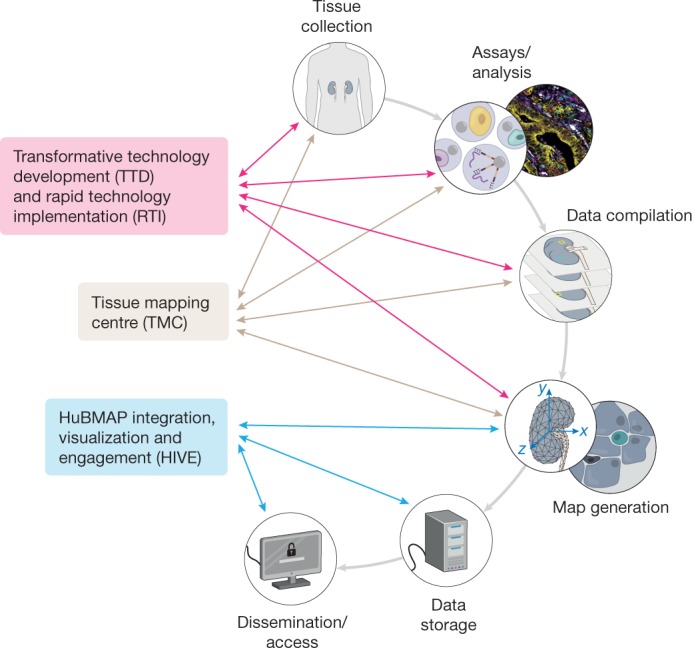
The HubMAP consortium. The TMCs will collect tissue samples and generate spatially resolved, single-cell data. Groups involved in TTD and RTI initiatives will develop emerging and more developed technologies, respectively; in later years, these will be implemented at scale. Data from all groups will be rendered useable for the biomedical community by the HuBMAP integration, visualization and engagement (HIVE) teams. The groups will collaborate closely to iteratively refine the atlas as it is gradually realized.

The HuBMAP consortium comprises members with diverse expertise (for example, molecular, cellular, developmental, and computational biologists, measurement experts, clinicians, pathologists, anatomists, biomedical and software engineers, and computer and data information scientists) and is organized into three components: (1) tissue mapping centres (TMCs); (2) HuBMAP integration, visualization and engagement (HIVE) collaborative components; and (3) innovative technologies groups (transformative technology development (TTD) and rapid technology implementation (RTI)) (Fig. 1). Throughout the program, HuBMAP will increase the range of tissues and technologies studied through a series of funding opportunities that have been designed to be synergistic with other NIH-funded and international efforts. In the later stages of HuBMAP, demonstration projects will be added to show the utility of the generated resources and, importantly, to engage the wider research community to analyse HuBMAP data alongside data from other programs or their own labs.

## Tissue and data generation

**Fig. 2:**
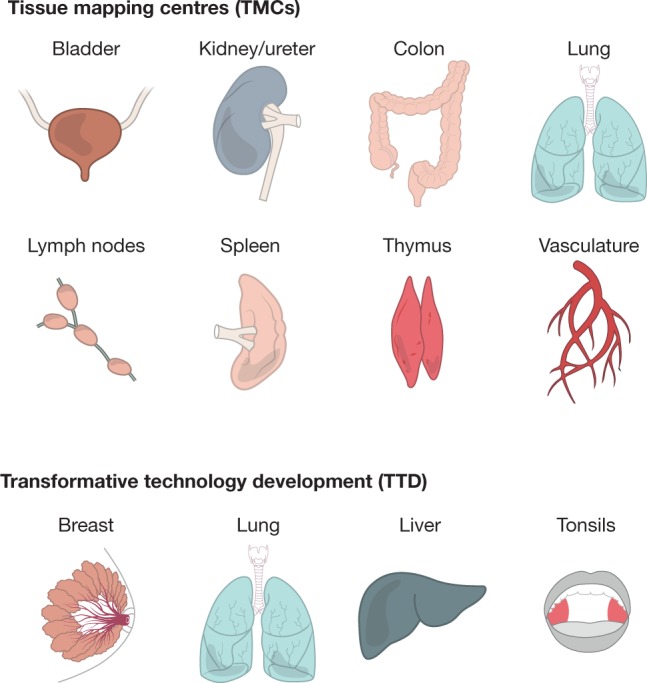
Key tissues and organs initially analysed by the consortium. Using innovative, production-grade (‘shovel ready’) technologies, HuBMAP TMCs will generate data for single-cell, three-dimensional maps of various human tissues. In parallel, TTD projects (and later RTI projects) will refine assays and analysis tools on a largely distinct set of human tissues. Samples from individuals of both sexes and different ages will be studied. The range of tissues will be expanded throughout the program.

The HuBMAP TMCs will collect and analyse a broad range of largely normal tissues, representing both sexes, different ethnicities and a variety of ages across the adult lifespan. These tissues (Fig. 2) include: (1) discrete, complex organs (kidney, ureter, bladder, lung, breast, small intestine and colon); (2) distributed organ systems (vasculature); and (3) systems comprising dynamic or motile cell types with distinct microenvironments (lymphatic organs: spleen, thymus, and lymph nodes). Tissue will be collected at precisely defined anatomical locations (when possible, photographically recorded) according to established protocols that preserve tissue quality and minimize degradation. Beyond meeting standard regulatory requirements, to the greatest extent possible, consent will be obtained so that the generated data is available for open-access data sharing (that is, public access without approval by data committees), to maximize their usage by the biomedical community.

**Fig. 3:**
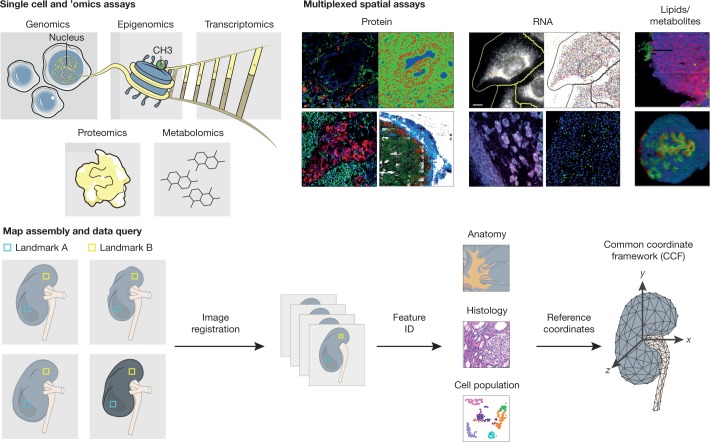
Map generation and assembly across cellular and spatial scales. HuBMAP aims to produce an atlas in which users can refer to a histological slide from a specific part of an organ and, in any given cell, understand its contents on multiple ’omic levels—genomic, epigenomic, transcriptomic, proteomic, and/or metabolomic. To achieve these ends, centres will apply a combination of imaging, ’omics and mass spectrometry techniques to specimens collected in a reproducible manner from specific sites in the body. These data will be then be integrated to arrive at a high-resolution, high-content three-dimensional map for any given tissue. To ensure inter-individual differences will not be confounded with collection heterogeneity, a robust CCF will be developed.

To achieve spatially resolved, single-cell maps, the TMCs will use a complementary, iterative, two-step approach (Fig. 3). First, ’omic assays, which are extremely efficient for data acquisition, will be used to generate global genome sequence and gene expression profiles of dissociated single cells or nuclei in a massively parallel manner. The molecular state of each cell will be revealed by single-cell transcriptomic^11^ and, in many cases, chromatin accessibility^12,13^ assays; imputation of transcription factor binding regions from the open chromatin data combined with the gene expression data will be used to explain the regulation of gene expression across the distinct cell types^14^. Second, spatial information (abundance, identities, and localization) will be acquired for various biomolecules (RNA^15^, protein^16^, metabolites, and lipids) in tissue sections or blocks, using imaging methodologies such as fluorescent microscopy (confocal, multiphoton, lightsheet, and expansion), sequential fluorescence in situ hybridization (seqFISH)^17,18^, imaging mass spectrometry^19,20^, and imaging mass cytometry (IMC)^21–24^. The extensive single-cell and nucleus profiles obtained will inform in situ modalities (for example, single-cell or nucleus RNA sequencing will be used to choose probes for RNA or proteins), which will provide spatial information for up to hundreds of molecular targets of interest. These data will allow the computational registration of cell-specific epigenomic or transcriptomic profiles to cells on a histological slide to reveal various microenvironmental states. They will potentially include information about protein localization to cytoplasm, nucleus, or cell surface; phosphorylation; complex assembly; extracellular environment; and cellular phenotype determined by protein marker coexpression. Registration and computational integration of complex imaging data will provide biological insights beyond any single imaging mode^19,25^. The powerful combination of single-cell profiling and multiplexed in situ imaging will provide a pipeline for constructing multi-omics spatial maps for the various human organs and their cellular interactions at a molecular level.

The TMCs will use complementary methods for data collection with an emphasis on processes to ensure the generation of high-quality data and standardized metadata annotations. Benchmarking, quality assurance and control standards, and standard operating procedures, where appropriate, will be developed for each stage of the methodological process and be made available to promote rigor, reproducibility and transparency. It is expected that quality assurance and control standards for both biospecimens and data will evolve as tissue collection, processing techniques, storage and shipping conditions, assays, and data-processing tools change, and as HuBMAP interacts and collaborates with other related efforts, as they have for other consortium projects^26–31^. Where possible, metadata related to preanalytical variables (for example, annotations and nomenclature) and technologies will be harmonized, and protocols and standards will be shared with the wider research community.

## Building an integrated tissue map across scales

The diversity of data generated by HuBMAP, ranging across macroscopic and microscopic scales (for example, anatomical, histological, cellular, molecular and genomic) and multiple individuals, is essential to its core mission. Exploring each of these valuable datasets collectively will yield an integrated view of the human body. Hence, HuBMAP will develop analytical and visualization tools to bridge spatial and molecular relationships in order to help to generate a high-resolution three-dimensional molecular atlas of the human body.

The volume of data generated and collected by HuBMAP will require the utilization, extension and development of tools and pipelines for data processing. While we expect that initial data-processing tools will be based on methods developed by consortium members, HuBMAP will also work with and incorporate algorithms developed by other programs and the wider research community to supplement, enhance or update its pipelines. To this end, HuBMAP will develop one or more portals tailored to emerging use cases identified through a series of user needs. These open source portals will use recognized standards and be interoperable with other platforms, such as the HCA Data Coordination Platform, making it possible to readily add, update, and use new software modules (for example, as with Dockstore^32^ and Toil^33^). The portion of HuBMAP data that will be open source can live on or be accessed from multiple platforms, enhancing its utility. This infrastructure will enable external developers to apply their codes, applications, open application programming interfaces, and data schema to facilitate customized processing and analysis of HuBMAP data in concert with other data sources. Furthermore, by actively working with other global and NIH initiatives, the consortium will seek to reduce the barriers to browsing, searching, aggregating, and analysing data across portals and platforms.

To fully integrate spatial and molecular data across individuals, HuBMAP will create a common coordinate framework (CCF) that defines a three-dimensional spatial representation, leveraging both an early consortium-wide effort to standardize technologies and assays using a single common tissue and the broader range of tissues of the human body analysed across multiple scales (whole body to single cells). This spatial representation will serve as an addressable scaffold for all HuBMAP data, enabling unified interactive exploration and visualization (search, filter, details on demand) and facilitating comparative analysis across individuals, technologies, and laboratories^34,35^. To achieve these objectives, HuBMAP envisions a strategy inspired by other tissue atlas efforts^36–38^ that leverages the identification of ‘landmark’ features, including key anatomical structures and canonical components of tissue organization (for example, epidermal boundaries and normally spatially invariant vasculature) that can be identified in all individuals. These landmarks will enable a ‘semi-supervised’ strategy for aligning and assembling an integrated reference, upon which HuBMAP investigators can impose diverse coordinate systems, including relative representations and zone-based projections. As one example, an open-source, computational histology topography cytometry analysis toolbox (histoCAT^39^) currently facilitates two-dimensional visualization and will soon also be applicable to three-dimensional reconstruction. Ontology-based frameworks will be explored in parallel to effectively categorize, navigate, and name multiscale data; synergies are expected between these two approaches. Whenever available, medical imaging, such as CT and MRI information, will serve as a basis for landmarking and constructing the CCF.

## Technology development and implementation

Quantitative imaging of different classes of biomolecule in the same tissue sample with high spatial resolution, sensitivity, specificity, and throughput is central to the development of detailed tissue maps. Although no single technique can fully address this challenge at present, the development and subsequent multiplexing of complementary capabilities provides a promising approach for accelerating tissue mapping efforts. The HuBMAP innovation technologies groups aim to develop several innovative approaches that will address the limitations of existing state-of-the-art techniques. For example, transformative technologies such as signal amplification by exchange reaction (SABER)^40,41^, seqFISH^18,42,43^, and Lumiphore probes^44^ will be refined to improve multiplexing, sensitivity, and throughput for imaging RNA and proteins across multiple tissues. Furthermore, new mass spectrometry imaging techniques will enable the quantitative mapping of hundreds of lipids, metabolites, and proteins from the same tissue section with high spatial resolution and sensitivity^45,46^. There is also scope within the program to develop and test new technologies. These efforts will benefit from the development of new computational tools and machine learning algorithms, optimized first from data generated from a common tissue during the pilot phase, for data integration across modalities.

## Challenges

Previous programs such as GTEx^5^ have faced the challenge of optimizing the collection, preservation, and processing of a wide variety of tissue types from multiple donors. However, one of the goals of HuBMAP, to generate comprehensive, interactive high-resolution maps using a wide variety of assays, introduces an added level of complexity. Mapping functionally important biomolecules, including some of which we may not even be aware and for which sensitive, specific, and high-throughput assays are still lacking, will require close attention. Moreover, the program will produce an unprecedented volume and diversity of datasets for comprehensive data capture, management, mining, modelling, visual exploration and communication. The integration of data from different modalities is required for generating robust maps; it will be necessary to develop corresponding analysis and interactive visualization tools to ensure that the data and atlas are accessible to the entire life-sciences community. Finally, given the enormity of a human atlas, HuBMAP faces the challenges of prioritization of tissues and technologies, sampling across tissues and donors, and optimally synergizing its efforts with international efforts. Determining the number of cells, fields of view, and samples needed to capture rare cell types, states or tissue structures is an important challenge, but can be tackled with adaptive power analyses, leveraging the growing amount of data available both within HuBMAP and from other consortia as well as individual groups.

## Resources and community engagement

HuBMAP is an important part of the international mission to build a high-resolution cellular and spatial map of the human body, and we are firmly committed to close collaboration and synergy with the aforementioned initiatives to build an easy-to-use platform and interoperable datasets that will accelerate the realization of a high-resolution human atlas. Shared guiding principles around open data, tools, and access will enable collaborative and integrated analyses of data produced by diverse consortia. To achieve this synergy, HuBMAP and other consortia will work together to tackle common computational challenges, such as cellular annotation, through formal and informal gatherings focused on addressing these problems, planned joint benchmarking and hands-on jamborees and workshops. Another example of the potential for close collaboration is in the study of the colon; multiple projects funded by HuBMAP, the Human Tumour Atlas Network, and the Wellcome Trust will be complemented by projects funded by the Leona M. and Harry B. Helmsley Charitable Trust. With projects focusing on partly distinct regions and diseases (for example, normal tissue, colon cancer, and Crohn’s disease), it will be important for all of the programs to ensure that data are collected and made available in a consistent manner, and HuBMAP will play an active part in such efforts. As a concrete next step, HuBMAP, in collaboration with other NIH programs, plans to hold a joint meeting with the Human Cell Atlas initiative to identify and work on areas of harmonization and collaboration during the spring of 2020. In parallel, HubMAP participants engage in the meetings and activities of other consortia, such as the Human Cell Atlas or the Human Tumour Atlas Network, thus forming tight connections. We have started a series of open meetings to develop the CCF, with the first of these recently held in collaboration with the Kidney Precision Medicine Program and focused on the kidney.

HuBMAP will provide capabilities for data submission, access, and analysis following FAIR (findable, accessible, interoperable, and reusable) data principles^47^. We will develop policies for prompt and regular data releases in commonly used formats, consistent with similar initiatives. We anticipate that the first round of data will be released in the summer of 2020, with subsequent releases at timely intervals thereafter. Robust metadata will comprise all aspects of labelling and provenance, including de-identified donor information (both demographic and clinical), details of tissue processing and protocols, data levels, and processing pipelines.

Indeed, engagement and outreach to the broader scientific community and other mapping centres is central to ensure that resources generated by HuBMAP will be leveraged broadly for sustained impact. To ensure that browsers and visualization tools from HuBMAP are valuable, the consortium will work closely with anatomists, pathologists, and visualization and user experience experts, including those with expertise in virtual or augmented reality. As described above, we expect that the diversity of normal samples included in this project will facilitate valuable comparative analyses, pinpointing how cells and tissue structures vary across individuals, throughout the lifespan, and in the emergence of dysfunction and disease. The program will build its resources with these use cases in mind and provide future opportunities, such as the demonstration projects, for close collaboration with domain experts. We also anticipate that these data will be highly useful for the generation of new biomedical hypotheses, tissue engineering, the development of robust simulations of spatiotemporal interactions, machine learning of tissue features, and educational purposes.

## Conclusions

Analogous to the release of the first human genome build, we anticipate that the first reference three-dimensional tissue maps will represent the tip of the iceberg in terms of their ultimate scope and eventual impact. HuBMAP, working closely with other initiatives, aspires to help to build a foundation by generating a high-resolution atlas of key organs in the normal human body and capturing inter-individual differences, as well as acting as a key resource for new contributions in the growing fields of tissue biology and cellular ecosystems. Given the focus of HuBMAP on spatial molecular mapping, the consortium will contribute to the community of efforts seeking similar goals, with a special emphasis on providing leadership in the development of analytical methods for its data types and for developing a common coordinate framework to integrate data. Ultimately, we hope to catalyse novel views on the organization of tissues, regarding not only which types of cells are neighbouring one another, but also the gene and protein expression patterns that define these cells, their phenotypes, and functional interactions. In addition to encouraging the establishment of intra- and extra-consortium collaborations that align with HuBMAP’s overall mission, we envision an easily accessible, publicly available user interface through which data can be used to visualize molecular landscapes at the single-cell level, pathways and networks for molecules of interest, and spatial and temporal changes across a given cell type of interest. Researchers will also be able to browse, search, download, and analyse the data in standard formats with rich metadata that, over time, will enable users to query and analyse datasets across similar programs.

Importantly, we believe that the project’s compilation of different types of multi-omic information at the single-cell level in a spatially resolved manner will represent an important step in the advancement of our understanding of human biology and precision medicine. These data have the potential to redefine types or subtypes of cells and their relationships within and between tissues beyond the traditional understanding that can be obtained through standard methods (for example, microscopy and flow cytometry). We hope this work will be part of a foundation that enables diagnostic interrogation, modelling, navigation, and targeted therapeutic interventions at such an unprecedented resolution to be transformative for the biomedical field.
